# Lymph node involvement by enteropathy-like indolent NK-cell proliferation

**DOI:** 10.1007/s00428-020-02892-8

**Published:** 2020-07-21

**Authors:** Jean-Louis Dargent, Nicolas Tinton, Mounir Trimech, Laurence de Leval

**Affiliations:** 1 Service d’Anatomie Pathologique, Institut de Pathologie et de Génétique (IPG), Gosselies, Belgium; 2 Service de Chirurgie, Grand Hôpital de Charleroi (GHDC), Charleroi, Belgium; 3 Institute of Pathology, Department of Laboratory Medicine and Pathology, Lausanne University Hospital and Lausanne University, Lausanne, Switzerland

**Keywords:** Indolent NK-cell lymphoproliferative disorder, NK-cell enteropathy, Lymphomatoid gastropathy, Lymph node, Gallbladder, Gastrointestinal

## Abstract

Natural killer (NK)-cell enteropathy (NKCE) and lymphomatoid gastropathy (LG) are closely related lymphoproliferative disorders (LPDs) composed of mature and Epstein–Barr virus (EBV)-negative NK-cells. Although these uncommon and indolent lymphoid proliferations mostly arise within the gastrointestinal (GI) tract as their designations implies, a few cases have been reported outside the GI tract. We hereby describe a unique case of lymph node infiltration by such EBV-negative NK-cell proliferation fortuitously found during routine examination of a gallbladder resected for biliary lithiasis. The histologic, phenotypic, and molecular features of this NK-cell proliferation, which were very similar if not identical to those previously reported in NKCE or LG, suggest that similar indolent EBV-negative NK-cell LPDs may also occasionally involve lymph nodes.

## Introduction

Several cases of a particular Epstein–Barr virus (EBV)-negative natural killer (NK)-cell proliferation occurring mainly in the gastrointestinal (GI) tract and following an indolent course have been recently reported [1–16]. The clinico-pathologic spectrum of this rare lymphoproliferative disorder (LPD), initially described as lymphomatoid gastropathy and more commonly referred to as NK-cell enteropathy (NKCE) [1–16], remains not fully characterized. Herein, we describe an EBV-negative NK-cell proliferation that was fortuitously found in the cystic duct lymph node of a cholecystectomy.

## Case report

**Fig. 1:**
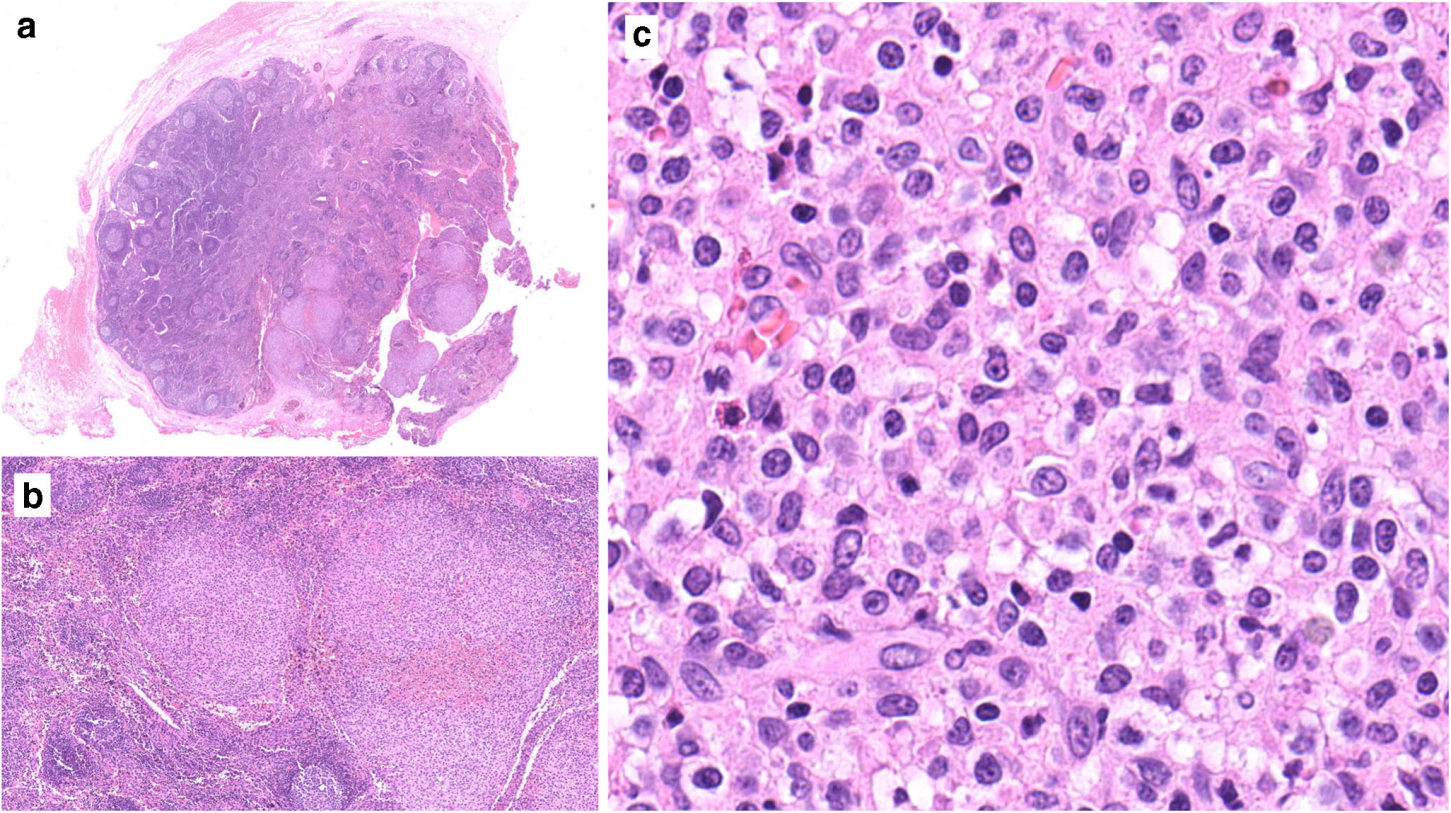
Histopathology of the lymph node. **a** Panoramic view showing partial replacement of the lymphoid tissue by relatively circumscribed nodules (lower right). Elsewhere, the nodal architecture is preserved with follicular hyperplasia. **b** The nodules are composed of monotonous and medium-sized lymphoid cells. **c** Cytological features at high power. Hematoxylin and eosin stain; original magnifications × 8, × 50, and × 400, respectively

**Fig. 2:**
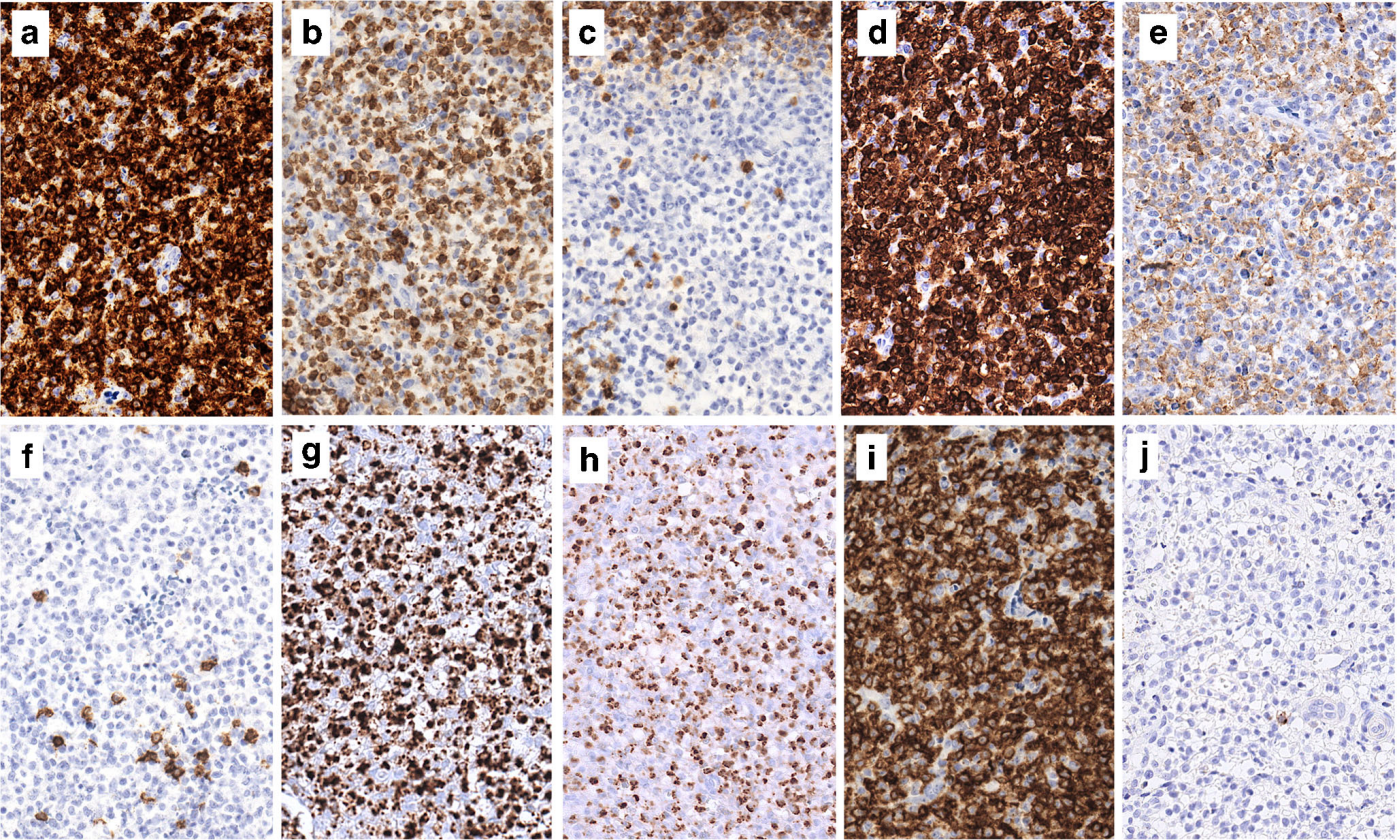
Immunophenotypic characteristics of the lymphoproliferation. The cells are positive for CD2 (a), cytoplasmic CD3 (b), CD7 (d), CD56 (i), perforin (g), and TIA1 (h). They are negative for CD5 (c), CD4 (e), CD8 (f), and CD57 (j). CD4 and CD8 stain a few reactive T cells or histiocytes. Immunoperoxidase; original magnification × 300

A 37-year-old Caucasian male with no previous medical history underwent laparoscopic cholecystectomy for symptomatic biliary lithiasis. The gallbladder contained gallstones, had a moderately thickened wall, and no obvious mucosal lesion. A 1.3-cm lymph node was adjacent to the cystic duct, which was free of stone. Histologically, the gallbladder showed hyperplastic and inflammatory changes consistent with xanthogranulomatous cholecystitis, and the cystic duct was normal. Beside benign reactive changes with B cell follicular hyperplasia, the lymph node was partially obliterated by a relatively circumscribed and vaguely nodular infiltrate of monotonous medium-sized cells with moderately abundant pale cytoplasm (Fig. 1a–b). No cytoplasmic granules were visible. Nuclei were round to oval with slightly irregular contours, somewhat open chromatin and inconspicuous nucleoli (Fig. 1c). There were rare apoptotic bodies, few mitoses, and scattered histiocytes. By immunohistochemistry, the pale cells were positive for CD2, cytoplasmic CD3 (± ), CD7, CD56, perforin, granzyme B (± ) and T-cell intracellular antigen (TIA)-1, and negative for T-cell receptor β (TCRβ), TCRδ, CD1a, CD4, CD5, CD8, CD10, CD20, CD23, CD30, CD34, CD57, CD79a, CD123, CXCL13, ICOS, PD1, S100, and myeloperoxydase (Fig. 2). The Ki-67 proliferation index was approximately 10%. EBV-encoded RNA (EBER) in situ hybridization (ISH) was negative. Polymerase chain reaction–based clonality analysis did not demonstrate clonal rearrangement of the TR genes (TRB and TRG). Next-generation sequencing (NGS) analysis using a panel of 26 genes designed for peripheral T-cell lymphomas (*ARID1A*, *ATM*, *BCOR*, *CARD11*, *CCR4*, *CD28*, *CTNNB1*, *DDX3X*, *DNMT3A*, *FYN*, *IDH2*, *IRF4*, *JAK1*, *JAK3*, *KMT2D*, *PIK3CD*, *PLCG1*, *PRKCB*, *RHOA*, *SETD2*, *STAT3*, *STAT5B*, *TET2*, *TNFRSF1B*, *TP53*, *VAV1*) disclosed a mutation involving the exon 4 of the *DNMT3A* gene (c.178-9C>T) with a variant allele fraction (VAF) of 46%, also described in the general population at a very low frequency and interpreted as a constitutional variant. FISH analysis using a *JAK2* break apart probe (9p24) showed a normal hybridization pattern.

Based on these findings, an “enteropathy-like” NK-cell lymphoproliferation was considered. An extensive clinical work-up, including peripheral blood analysis, computed tomography (CT) scan, positron emission tomography (PET) scan, esophagogastroduodenoscopy, and colonoscopy, did not demonstrate any abnormal finding. Multiple biopsies from the stomach, duodenum, ileum, and colon showed essentially normal histology. PET scan disclosed a small thyroid nodule, benign by fine needle aspiration cytology. No further therapy was given. The patient is still asymptomatic 18 months later.

## Discussion

Except for the unusual location within a lymph node, the features of this EBV-negative NK-cell proliferation are very similar to those described in NKCE, of which forty-seven cases have been reported [1–16]. The clinical features of these uncommon lymphoid proliferations, which share some features with indolent T-cell LPDs of the GI tract [17, 18], are summarized in Table 1. There were 19 men and 28 women ranging in age from 9 to 76 years, only four patients were younger than 30 years, and the median age was 57.5 years [1–16]. With the exception of three cases [11, 14, 16], all patients were diagnosed with GI involvement [1–16]. Twenty-eight cases occurred in the stomach, duodenum, or in several upper GI sites; seven cases involved the lower GI tract; and in nine cases there was concomitant involvement of the upper and lower GI mucosa [1–16]. In roughly half of the cases (21/44) the patients had no specific complaints and the lesions were fortuitously discovered by endoscopy performed for check-up or screening [1–16]. Conversely, in 23/44 cases various symptoms were recorded, mostly vague or non-specific abdominal or epigastric pain or discomfort, sometimes diarrhea or weight loss, for which clinical workup and endoscopic investigations eventually led to the diagnosis [1–16]. At endoscopy, the altered mucosa often consists of erythematous, flat or polypoid lesions, superficial erosions, or ulcers [1–16]. The clinical course is particularly indolent, with spontaneous resolution or persistence despite various kinds of therapies, for up to several years in some patients [1–16]. Unlike indolent T-cell LPDs of the GI tract, progression to a more aggressive disease or nodal involvement has not yet been documented, although imaging studies showed enlarged regional lymph nodes in some patients [4, 9]. The cases reported outside the GI tract were incidental microscopic discoveries during gallbladder examination in two patients (33- and 65-year-old) who underwent cholecystectomy for chronic cholecystitis or biliary colic, and a symptomatic vaginal mass in a 34-year-old woman [11, 14, 16]. The case reported here is the first example of nodal involvement by an indolent EBV-negative NK-cell LPD similar to NKCE or LG. Interestingly, the involved lymph node was adjacent to the gallbladder, suggesting that outside of the GI tract, the biliary region may be a site of predilection for this disorder, which may possibly originate from a peculiar tissue-specific NK-cell subset, as has been proposed in some studies [3, 5, 9, 13].

**Table 1:** Indolent NK-cell enteropathy and enteropathy-like indolent NK cell lymphoproliferations: summary of clinical features

Study	Number of cases	Sex	Age	Presentation	Localization of disease	Treatment	Follow-up, status
This study	1	M	37	Biliary lithiasis	Lymph node (cholecystectomy)	Observation	18 mo, AW
Takeuchi et al. [2]	10	5M:5F	46–75	Asymptomatic, follow-up of gastric cancer (3) cancer screening (7)	Upper GI (stomach) (10)	Observation (8/10) partial or total gastrectomy (2/10)	18–60 mo, AWD (3) 12–145 mo, AW (7)
Vega et al. [1] and ^a^Mansoor et al. [3]	1 and 8	2M:6F	27–68	Abdominal symptoms (6), asymptomatic and cancer screening (2)	Upper GI (stomach ±duodenum) (2) Lower GI (colon) (3) Upper and lower GI (3)	Observation (4) gluten- and lactose-free diet (1) Chemotherapy (CHOP) ± bone marrow transplantation (3)	22–120 mo, AW (8)
McElroy et al. [4]	1	F	52	Diarrhea, weight loss, abdominal pain	Upper and lower GI (stomach, small intestines, colon)	Gluten-free diet, chemotherapy (CHOP), steroids	8 years, AWD
Yamamoto et al. [5]	1	M	70	Cancer screening	Upper GI (stomach)	Observation	NA, AW
Tanaka et al. [6]	1	M	50	Check-up	Upper GI (stomach)	*H. pylori* eradication	3 years, ANED
Terai et al. [7]	1	F	57	Check-up	Upper GI (stomach)	*H. pylori* eradication	12 mo, ANED
Ishibashi et al. [8]	1	F	71	Epigastric discomfort	Upper GI (stomach)	Observation	11 mo, ANED
Koh et al. [9]	1	M	14	Vomiting, abdominal discomfort, diarrhea	Upper and lower GI (esophagus, stomach, small intestines, colon)	Observation	40 mo, AWD
Takata et al. [10]	6	3M:3F	49–72	Epigastric pain (2) or no symptoms (4)	Upper GI (stomach) (6)	Surgical resection (1)	80 mo, AWD (1) 6–57 mo, ANED (5)
Hwang et al. [11]	1	F	33	Abdominal pain, GI symptoms	Gallbladder (cholecystectomy)	Observation	36 mo, ANED
Isom et al. [12]	1	F	69	Abdominal pain, GI symptoms	Upper GI (stomach and duodenum)	*H. pylori* eradication	10 years, AWD
Wang et al. [13]	1	M	58	Asymptomatic, cancer screening	Lower GI (colonic polyp)	None	6 mo, ANED
Xia et al. [14]	4	3M:1F	45–68	Anemia (1), biliary colic (1), routine colonoscopy (1), abdominal symptoms and suspected inflammatory bowed disease (1)	Lower GI (small intestines or colon) (2) Gallbladder (cholecystectomy) (1) Upper and lower GI (1)	Observation	2–33 mo, AW (3) 124 mo, AWD (1)
^a^Xiao W [15]	10	3M:7F	9–76	GI symptoms (7), asymptomatic (2)	Upper GI (6) upper and lower GI (3) Lower GI (1)	Observation (3) *H. pylori* eradication (1) Methotrexate (1)	24–95 mo, A (4) 48–96 mo, AWD (4)
Krishnan et al. [16]	1	F	34	Vaginal mass during early pregnancy	Vagina (biopsy and resection)	Observation	24 mo, ANED

*F* female, *M* male, *GI* gastrointestinal, *NA* not available, *A* alive, *AW* alive and well, *AWD* alive with persistent disease, *ANED* alive with no evolutive disease

^a^These studies included the patient initially described by Vega et al. [1]

NKCE is usually composed of medium to large-sized lymphoid cells that contain variable amounts of pale cytoplasm, nuclei with slightly irregular contours, open chromatin, and inconspicuous nucleoli [1–16]. In most cases, atypical cells diffusely infiltrate the lamina propria of the mucosa [1–16]. Occasionally, there may be some spilling into the glandular epithelium. Extension into the submucosa was reported in some cases [1–16]. Importantly, there is no marked cellular atypia, no significant mitotic activity, and no angiocentricity, angiodestructive growth or necrosis [1–16]. Immunophenotypic features of the reported cases show a very homogeneous profile consistent with mature NK cell, including expression of cytoplasmic CD3, CD7, CD56, and cytotoxic granules-associated molecules in virtually all cases [1–16]. CD2 expression is detected in a subset of the cases (68%), a few CD8+ cases have been reported (4/43, 9%) [9, 10, 12], while CD4, CD5, and TCR isoforms (β or γ chains) are consistently negative. Ki-67 proliferation index is typically reported as low or quantified lower than 50%, but in 5/26 cases (19%) a higher fraction of cycling cells was reported, up to 90%. EBER ISH was negative in all cases, and no clonal rearrangement of the TR genes was found in any of the cases where clonality was investigated [1–16].

Some studies have suggested that an abnormal immune response, possibly related to yet unknown antigen(s), could underline NKCE pathogenesis [3, 5, 9, 13, 16]. In one patient with high titers of antigliadin antibodies, the lesions regressed significantly under gluten-free and lactose-free diet [1]. Several gastric cases had concomitant *Helicobacter pylori* infection [2, 6–8, 10, 12]. However, overall, no firm relationship with a specific factor or condition could be demonstrated [1–16]. In a recent study using a large hematology-oriented NGS panel, a somatic *JAK3* mutation consisting of a small in frame deletion in exon 12 was identified in 3/10 patients with NKCE [15]. Other non-recurrent mutations involving *PTPRS*, *AURKB*, *AXL*, *ERBB4*, *IGF1R*, *PIK3CB*, *CUL3*, *CHEK2*, *RUNX1T1*, *CIC*, *SMARCB1*, and *SETD5* were found in seven cases [15]. In a few cases subject to extensive genetic testing including 3/10 NKCE and the vaginal mass recently reported [15, 16], however, no mutation was identified. In our patient, NGS analysis of 26-genes T-cell lymphoma panel found no pathogenic mutation and in particular no *JAK3* deletion, but one cannot exclude genetic aberration(s) in other genes not tested in this panel. Thus, indolent NK-cell LPDs may represent a spectrum ranging from polyclonal to clonal, the latter related to mutations promoting the clonal expansion of NK cells, notably those deregulating the JAK/STAT pathway which represent oncogenic drivers in a variety of other NK/T-cell neoplasms.

Due to significant prognostic differences and therapeutic implications, indolent NK-cell LPDs must be distinguished from other mature NK-cell neoplasms like aggressive NK-cell leukemia and extranodal NK/T-cell lymphoma, nasal-type. Marked cellular atypia, significant mitotic activity, cellular necrosis, angiocentricity, or angiodestructive pattern of growth and positive EBER ISH are features of aggressive NK-cell disorders and clues against NKCE [1–16]. Distinction from the exceptional cases of EBV-negative NK-cell lymphoma may be more challenging due to EBV-negativity, but those cases as the one reported by Koo et al. in the small bowel show clearly malignant features [17]. Because of clear cells and possible epitheliotropism, NKCE may first elicit consideration of a marginal zone B cell lymphoma or monomorphic epitheliotropic intestinal T-cell lymphoma (MEITL). Even after immunophenotyping, the distinction with MEITL may remain difficult, given expression of antigens common to T and NK cell lineages and strong CD56 positivity in both conditions [14]. However, MEITL typically presents as a tumor mass, harbors clonal TR gene rearrangements, and recurrent *SETD2* alterations, not found in NKCE [18].

In conclusion, we describe herein a case of nodal EBV-negative NK-cell proliferation, akin to NKCE. This observation further expands the spectrum of NKCE outside the GI tract. Awareness of pathologic features of these underrecognized LPDs is advisable, in order to avoid overdiagnosis and inappropriate treatment.
